# Dezesseis Anos de Transplante Cardíaco em Coorte Aberta no Brasil: Análise de Sobrevivência de Pacientes em Uso de Imunossupressores

**DOI:** 10.36660/abc.20200117

**Published:** 2021-04-08

**Authors:** Natália Cristina Cardoso Freitas, Mariangela Leal Cherchiglia, Charles Simão, Juliana Alvares-Teodoro, Francisco de Assis Acurcio, Augusto Afonso Guerra

**Affiliations:** 1 Universidade Federal de Minas Gerais Faculdade de Farmácia Departamento de Farmácia Social Belo HorizonteMG Brasil Universidade Federal de Minas Gerais - Faculdade de Farmácia - Departamento de Farmácia Social, Belo Horizonte, MG - Brasil.; 2 Universidade Federal de Minas Gerais Faculdade de Medicina Departamento de Medicina Preventiva e Social Belo HorizonteMG Brasil Universidade Federal de Minas Gerais - Faculdade de Medicina - Departamento de Medicina Preventiva e Social, Belo Horizonte, MG - Brasil.; 3 Universidade Federal de Minas Gerais Faculdade de Medicina Departamento de Cirurgia Belo HorizonteMG Brasil Universidade Federal de Minas Gerais - Faculdade de Medicina - Departamento de Cirurgia, Belo Horizonte, MG – Brasil.

**Keywords:** Transplante Cardíaco/tendências, Ciclosporina/uso terapêutico, Sobrevivência, Imunossupressores, Epidemiologia

## Abstract

**Fundamento:**

O transplante cardíaco é a principal alternativa terapêutica para pacientes com insuficiência cardíaca avançada. Diversos fatores de risco influenciam a sobrevivência desses pacientes, entretanto, poucos estudos acerca do tema estão disponíveis no Brasil.

**Objetivos:**

Analisar a sobrevivência de pacientes transplantados cardíacos pelo Sistema Único de Saúde no Brasil entre 2000-2015.

**Métodos:**

Trata-se de uma coorte não concorrente, aberta, de pacientes transplantados cardíacos no Brasil. A probabilidade acumulada de sobrevivência foi estimada por Kaplan-Meier e a comparação entre as curvas realizada pelo Teste de Log-Rank. O modelo de Cox foi utilizado para calcular o Hazard-Ratio (HR). As análises foram realizadas ao nível de 95% de confiança.

**Resultados:**

A mediana de sobrevivência do transplante cardíaco no Brasil no período foi 8,3 anos. Cada ano adicional na idade do receptor, a ocorrência de infecções e a realização do procedimento cirúrgico na região Sul relacionaram-se ao maior risco de perda do enxerto. Maior proporção de uso dos imunossupressores micofenolato e azatioprina atuou como fator protetor.

**Conclusões:**

As análises realizadas fornecem a primeira informação quanto ao tempo de sobrevivência mediana do transplante cardíaco no Brasil. A diferença observada entre as regiões pode estar relacionada aos diferentes protocolos de tratamento adotados no país, principalmente no início dos anos 2000. A proporção de uso de micofenolato e azatioprina como fator protetor sugere que, apesar de não haver diferença entre as estratégias terapêuticas, o uso desses medicamentos pode favorecer a sobrevida de determinados pacientes. O estudo apresenta dados epidemiológicos robustos e importantes para a saúde pública.

## Introdução

O transplante cardíaco (TC) é a principal alternativa terapêutica para pacientes com insuficiência cardíaca (IC) avançada, refratária às intervenções clínicas e cirúrgicas otimizadas, tendo como principal objetivo melhorar a sobrevida e qualidade de vida desses indivíduos.^1^ Uma vez transplantado, o paciente requer a utilização prolongada de esquemas farmacológicos de imunossupressão para manutenção do transplante. E, embora as recomendações atuais permitam a combinação e a utilização de diversos medicamentos, os esquemas tríplices, contendo corticosteroide, inibidor de calcineurina e agente antiproliferativos, continuam sendo amplamente referenciados pelas diretrizes e utilizados nos serviços de saúde.^2^

Desde a introdução da ciclosporina na década de 1980, o número de transplantes cardíacos e as taxas de sobrevida têm aumentado progressivamente em todo o mundo. Contudo, diversos fatores de risco permanecem influenciando a sobrevivência no TC, dentre eles: características demográficas do receptor e do doador, variáveis clínicas como a causa da IC, estratégias terapêuticas de manutenção adotadas e a incidência de complicações pós-transplante.^3^^,^^4^

O Brasil possui um dos maiores sistemas públicos de transplantes do mundo, sendo quase a totalidade desses procedimentos realizados pelo Sistema Único de Saúde (SUS). O país é atualmente destaque na América Latina e considerado uma referência no transplante de coração na doença de Chagas.^5^ O TC e o acompanhamento dos pacientes transplantados, desde o manejo pré-operatório até a disponibilização dos imunossupressores pós transplante, está entre as trinta terapias mais onerosas disponíveis à população brasileira por meio do SUS, sendo o sistema responsável por cerca de 96% dos TC realizados no país.^6^

Diferentemente de outros países, contudo, poucos estudos sobre a sobrevivência no TC estão disponíveis no Brasil. Os dados são escassos e difusos, de modo que não existem informações robustas no que se refere à sobrevida do enxerto e seus respectivos fatores de risco na população brasileira. Nesse contexto, o objetivo do presente estudo é analisar a sobrevivência de pacientes transplantados cardíacos pelo SUS no Brasil entre os anos de 2000-2015, com registro de uso de esquemas de imunossupressão.

## Métodos

Trata-se de uma coorte não concorrente, aberta, de pacientes submetidos ao TC pelo SUS no Brasil. Essa coorte foi construída por meio de um pareamento determinístico-probabilístico – método utilizado para integrar e unificar dados de um mesmo paciente, provenientes de diferentes sistemas de informação em saúde – das bases de dados administrativos do SUS: Sistema de Informações Hospitalares do SUS (SIH/SUS), Sistema de Informações Ambulatoriais (SIA/SUS) e Sistema de Informação de Mortalidade (SIM).^7^ Foram incluídos todos os pacientes transplantados cardíacos pelo SUS, no período de 01/01/2000 a 31/12/2014. A data de registro do transplante foi definida como a data de entrada na coorte e um período mínimo de 12 meses de seguimento foi estabelecido, de modo que o término do acompanhamento se deu em 31/12/2015. Para os pacientes incluídos nessa primeira fase foi realizada, inicialmente, a avaliação da sobrevivência geral no TC no Brasil.

Em seguida, foi extraída uma coorte de pacientes adultos, para a qual foram aplicados os seguintes critérios de exclusão à coorte anterior: idade inferior a 18 anos; indivíduos que se submeteram a transplante múltiplo; indivíduos cujo primeiro registro na coorte foi um retransplante; e indivíduos que não tiveram registro de uso de medicamento imunossupressor na base de dados.

### Análise estatística

Foi realizada a análise descritiva das variáveis utilizadas no estudo e a análise de sobrevivência.

Análise estatística descritiva foi realizada para todas as variáveis, sendo as categóricas analisadas por meio de distribuição de frequências absolutas e relativas: sexo, faixa etária, região em que o transplante foi realizado, diagnóstico primário da IC, tempo mediano de doença cardiovascular (DCV) antes do transplante ≥ 17 meses, comorbidades/complicações desenvolvidas após o transplante e esquema terapêutico de imunossupressão. A proporção de tempo de uso de cada medicamento, até o evento ou censura para cada paciente na coorte, foi analisada pela mediana e intervalo interquartil, assim como para a idade geral da população adulta tais medidas também foram apresentadas.

Para as análises de sobrevivência foram utilizados os seguintes parâmetros: o evento, definido como a perda do enxerto e representado, neste estudo, pela ocorrência de óbito ou retransplante; a censura informativa, considerada a data do último registro referente à imunossupressão; e a censura à direita, ou seja, a interrupção do estudo, representada pela data de término do acompanhamento (31/12/2015).

O estimador Kaplan-Meier foi utilizado para determinar a probabilidade acumulada de sobrevivência do enxerto dos pacientes incluídos em ambas as coortes. A diferença entre as curvas foi comparada por meio do Teste de *Log-Rank*. As variáveis foram avaliadas individualmente, a fim de se verificar o efeito de cada uma na sobrevivência, aquelas que apresentaram valor de p<0,20 foram incluídas no modelo final, multivariado. O modelo semiparamétrico de riscos proporcionais, modelo de Cox, foi utilizado para calcular o *Hazard-Ratio* (HR) destas análises uni e multivariadas. A análise de resíduos de Schoenfeld foi utilizada para verificar o ajuste e a proporcionalidade de riscos do modelo final. Todas as análises foram realizadas considerando o intervalo de confiança a 95%.

As análises estatísticas foram realizadas no software “R”, versão 3.6.0, da R Foundation for Statistical Computing.

Esse estudo obteve parecer favorável do Comité de Ética em Pesquisa da Universidade Federal de Minas Gerais (CAAE - 16334413.9.0000.5149).

## Resultados

**Figura 1:**
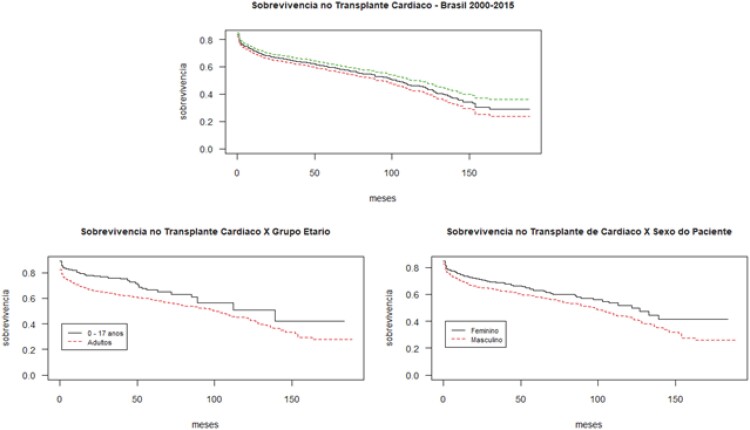
Sobrevivência do enxerto dos pacientes transplantados cardíacos no Brasil entre 2000-2015. Nota: As linhas tracejadas em verde e vermelho no primeiro gráfico desta figura representam, respectivamente, os limites superior e inferior do intervalo de confiança (95%).

Um total de 2.197 pacientes transplantados cardíacos no Brasil, entre os anos de 2000-2014, foram identificados, em sua maioria do sexo masculino (70,7%), dos quais 88,9% (n=1954) eram adultos e 11,1% (n=243) menores de 17 anos. A análise da sobrevivência da coorte demonstrou taxas de 70,9% (69,0 – 72,9) em um ano, 59,5% (57,1 – 61,9) em cinco anos, chegando a 45,1% (41,4 – 49,1) em dez anos e 29,1% (23,6 – 35,9%) no final do seguimento (13,6 anos). A mediana de sobrevivência do TC no país nesse período foi alcançada em 8,3 anos (Figura 1).

Ao comparar os grupos etários – adultos e menores de 17 anos – observou-se uma diferença estatisticamente significante entre os mesmos (p=0,003), tendo os adultos uma sobrevivência ligeiramente menor. O mesmo pode ser verificado na comparação por sexo, em que os indivíduos do sexo masculino apresentam menor sobrevida após o TC (p=0,01).

Como objeto principal desse estudo, selecionou-se a coorte de pacientes adultos (maiores de 18 anos), inicialmente com 1.954 pacientes, da qual foram excluídos: cinco pacientes cuja entrada na coorte foi proveniente de um retransplante cardíaco, seis que realizaram transplante múltiplo e 740 pacientes que não possuíam registro de uso de medicamentos na base de dados, para os quais verificou-se que 456 tinham registro de óbito e os 284 restantes, acredita-se que tenham obtido os imunossupressores por meio da saúde suplementar e/ou desembolso próprio. Desta forma, 1.203 pacientes foram incluídos no estudo.

A mediana de sobrevivência para essa população – pacientes adultos em uso de esquemas de imunossupressão – foi de 11,1 anos. As taxas de sobrevida em um, cinco e dez anos foram 89,8% (88,1 – 91,6), 75,9% (73,1 – 78,8) e 57,0% (52,1 – 62,3), respectivamente.

Dos 1.203 pacientes incluídos no estudo, a maioria era do sexo masculino (73,2%), com uma idade mediana de 48 anos (38 – 56). Para 69,1% desses pacientes (n=831) não foi possível identificar exatamente qual foi a condição primária que levou ao desenvolvimento da IC, visto que o primeiro registro na base de dados foi a própria condição. As cardiopatias isquêmicas aparecem como a segunda causa mais relatada, correspondendo a 14,1%, enquanto outras causas e malformações congênitas foram as causas menos frequentes, tendo registro para 0,3 e 1,7% dos indivíduos, respectivamente (Tabela 1).

**Tabela 1:** Características demográficas da população do estudo

Característica	Total (n = 1203)	
	n	%
**Região do Transplante**		
Centro Oeste	43	3,6
Nordeste	222	18,5
Norte	8	0,7
Sudeste	672	55,9
Sul	258	21,4
**Sexo**		
Feminino	323	26,8
Masculino	880	73,2
**Faixa Etária (anos)**		
18 - 25 anos	54	4,5
26 - 35 anos	179	14,9
36 - 45 anos	271	22,5
46 - 55 anos	392	32,6
56 - 65 anos	278	23,1
> 65 anos	29	2,4
**Causa da Insuficiência Cardíaca**		
Cardiomiopatias	76	6,3
Cardiopatias indefinidas	831	69,1
Cardiopatia isquêmica	170	14,1
Malformações congênitas	20	1,7
Outras doenças cardíacas	4	8,5
Outras causas	102	0,3
**Tempo mediano de Doença Cardiovascular anterior**		
Tempo mediano menor ou igual a 17 meses	434	36,1
Tempo mediano maior que 17 meses	427	35,5
**Comorbidades/complicações pós transplante**		
Dislipidemia	48	4,0
Hipertensão arterial	134	11,1
Infecções	45	3,7
Neoplasias	11	0,9
**Eventos**		
Censura	891	74,1
Óbito	307	25,5
Retransplante	5	0,4

Poucos registros foram verificados acerca de comorbidades ocorridas após o transplante, dentre elas: hipertensão arterial (11,1%), infecções (3,7%), dislipidemia (4,0%) e neoplasias (0,9%) (Tabela 1). Não foram encontrados registros de diabetes, doença renal crônica e osteoporose.

**Figura 2:**
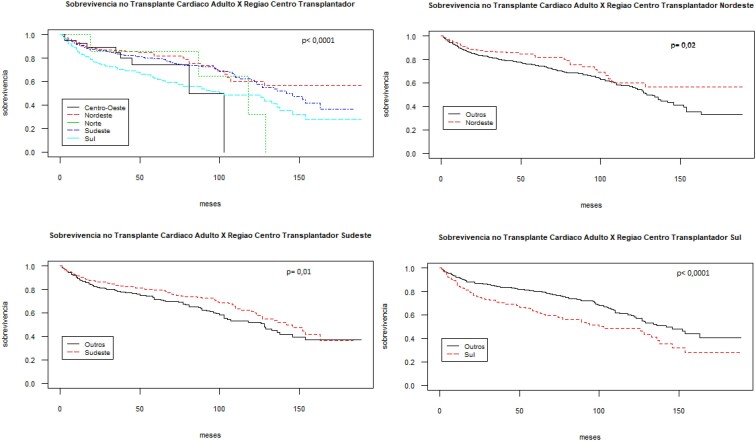
Sobrevivência do enxerto dos pacientes adultos transplantados cardíacos no Brasil entre 2000-2015 por região.

A maior parte das cirurgias de transplante foi realizada nas regiões Sudeste (55,9%), Sul (21,5%) e Nordeste (18,5%) (Tabela 1), tendo sido observada diferenças estatisticamente significativas na sobrevivência dos pacientes submetidos ao procedimento nessas regiões. Nordeste e Sudeste apresentaram uma melhor sobrevida (p= 0,02 e p= 0,01, respectivamente), ao passo que a região Sul apresentou taxas inferiores à média nacional (p<0,0001). As regiões Centro-Oeste e Norte não exibiram diferenças significativas (Figura 2).

No que se refere ao uso de esquemas imunossupressores, verificou-se que a ciclosporina associada ao micofenolato foi o esquema terapêutico de primeira escolha utilizado pela maioria destes pacientes (58,4%), seguido por micofenolato em monoterapia (18,4%) e pela associação entre ciclosporina e azatioprina (11,9%). A escolha de tacrolimo como inibidor de calcineurina na primeira linha de tratamento foi incipiente neste período, sendo que somente 3,3% dos indivíduos iniciaram seu tratamento com o mesmo; ao passo que o micofenolato foi o agente antiproliferativo mais utilizado, estando presente em cerca de 81% dos esquemas terapêuticos (Tabela 2).

**Tabela 2:** Esquemas imunossupressores de primeira escolha utilizados pela população do estudo

Principais esquemas Imunossupressores Início da Coorte	N	%	%acumulado*
Ciclosporina + Micofenolato	702	58,4%	58,4%
Micofenolato (monoterapia)	221	18,4%	76,7%
Ciclosporina + Azatioprina	143	11,9%	88,6%
Ciclosporina (monoterapia)	52	4,3%	92,9%
Micofenolato + Tacrolimo	34	2,8%	95,8%
**Subtotal**	**1.152**	**95,8%**	**95,8%**
**Outros esquemas imunossupressores Início da Coorte**			
Azatioprina (monoterapia)	22	1,8%	97,6%
Micofenolato + Sirolimo	15	1,2%	98,8%
Tacrolimo (monoterapia)	3	0,2%	99,1%
Azatioprina + Ciclosporina+ Micofenolato	2	0,2%	99,3%
Azatioprina +Tacrolimo	2	0,2%	99,4%
Ciclosporina + Sirolimo	2	0,2%	99,6%
Sirolimo (monoterapia)	2	0,2%	99,8%
Azatioprina +Sirolimo	1	0,1%	99,8%
Micofenolato + Ciclosporina+ Sirolimo	1	0,1%	99,9%
Micofenolato + Sirolimo + Tacrolimo	1	0,1%	100,0%
**Subtotal**	**51**	**4,2%**	**100%**
**Total**	**1.203**	**100%**	**100%**

* somatório dos percentuais de cada esquema linha a linha.

Ao estratificar o uso dos esquemas imunossupressores de primeira escolha por região, observou-se que o uso da associação ciclosporina e azatioprina foi proporcionalmente maior na região Sul do país (27,9%), representando cerca de 2,3 vezes a média nacional. Por outro lado, a associação ciclosporina e micofenolato foi o esquema terapêutico mais utilizado em todas as regiões (Tabela 3).

**Tabela 3:** Esquemas imunossupressores de primeira escolha utilizados pela população do estudo estratificado por região

Região do Centro Transplantador	Azatio+Ciclos	Ciclos (monoterapia)	Ciclos+Micofe	Micofe (monoterapia)	Micofe+Tacrol	Outros esquemas	Total Geral
	n (%)						
Centro Oeste	5(11,6)	1(2,3)	17(39,5)	8(18,6)	1(2,3)	11(25,6)	43(100,0)
Nordeste	12(5,4)	7(3,1)	133(59,9)	58(26,1)	1(0,4)	11(4,9)	222(100,0)
Norte	0(0,0)	0(0,0)	8(100,0)	0(0,0)	0(0,0)	0(0,0)	8(100,0)
Sudeste	54(8,0)	18(2,7)	410(61,0)	138(20,5)	28(4,2)	24(3,6)	672(100,0)
Sul	72(27,9)	26(10,1)	134(51,9)	17(6,6)	4(1,5)	5(1,9)	258(100,0)
Total Geral	143(11,9)	52(4,3)	702(58,4)	221(18,4)	34(2,8)	51(4,2)	1203(100,0)

Azatio: azatioprina; Ciclos: ciclosporina; Micofe: micofenolato; Tacrol: tacrolimo.

**Figura 3:**
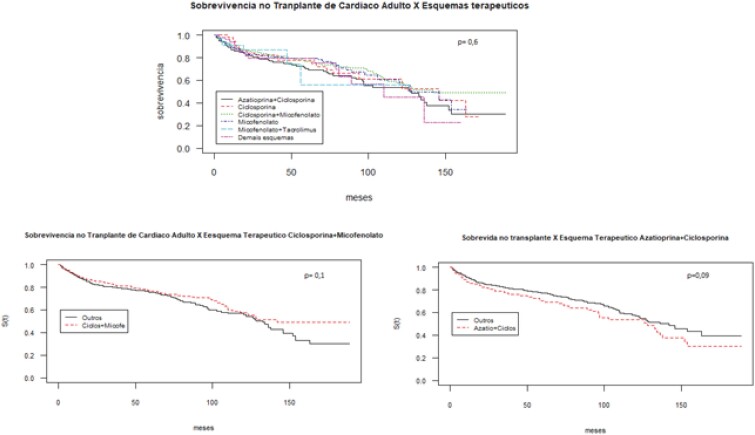
Sobrevivência do enxerto dos pacientes adultos transplantados cardíacos no Brasil entre 2000-2015 por esquemas de imunossupressão.

Ao avaliar a sobrevivência dos pacientes de acordo com o esquema imunossupressor inicialmente utilizado, não se observou diferença estatisticamente significante (p=0,6) (Figura 3).

A mediana de proporção de uso dos imunossupressores no tempo foi de 83,3% para micofenolato (65,7 – 95,2), 71,1% para ciclosporina (38,5 – 91,7), 38,2% para azatioprina (11,5 – 66,8), 26,0% para tacrolimo (8,3 – 47,2), 15,0% para sirolimo (4,8 – 34,7) e 7,1% para everolimo (2,4 – 28,8).

Na análise univariada dos potenciais fatores de risco para a sobrevivência do enxerto, verificou-se maior risco associado ao sexo masculino (HR = 1,342; IC 95% 1,02 – 1,767), ao ano adicional à idade do receptor (HR = 1,01; IC 95% 1,003 – 1,023), à realização da cirurgia região Sul do Brasil (HR = 1,784; IC 95% 1,407 – 2,262), ao tempo mediano de DCV antes do transplante superior a 17 meses (HR = 1,389; IC 95% 1,067 – 1,807), ao desenvolvimento de infecções após o transplante (HR = 1,702; IC 95% 1,012 – 2,861) e a uma proporção maior de uso de azatioprina ao longo do seguimento (HR = 1,769; IC 95% 1,125 – 2,783) (Tabela 4).

**Tabela 4:** Hazard-Ratio para perda do enxerto – análise univariada

Variável	Total (n = 1203)	
	HR (IC 95%)	p
**Região do Transplante**		
Centro Oeste	1,128 (0,580 – 2,194)	0,7
Nordeste	0,688 (0,499 – 0,950)	0,02
Norte	1,489 (0,555 – 3,997)	0,4
Sudeste	0,758 (0,607 – 0,945)	0,01
Sul	1,784 (1,407 – 2,262)	<0,001
Sexo masculino	1,342 (1,019 – 1,767)	0,04
Idade	1,013 (1,003 – 1,023)	0,01
**Causa da Insuficiência Cardíaca**		
Cardiomiopatias	0,962 (0,617 – 1,498)	0,9
Cardiopatias indefinidas	1,144 (0,899 – 1,457)	0,3
Cardiopatia isquêmica	0,950 (0,681 – 1,323)	0,8
Malformações congênitas	0,349 (0,087 – 1,404)	0,1
Outras doenças cardíacas	3,67 (0,912 – 14,77)	0,05
Outras causas	0,863 (0,593 – 1,256)	0,4
Tempo mediano de DCV antes do transplante	1,389 (1,067 – 1,807)	0,01
**Ocorrência de comorbidades pós transplante**		
Dislipidemia	0,919 (0,473 – 1,786)	0,8
Hipertensão arterial	1,270 (0,896 – 1,800)	0,2
Infecções	1,702 (1,012 – 2,861)	0,04
Neoplasias	1,363 (0,339 – 5,490)	0,7
**Esquemas imunossupressores de primeira escolha**		
Ciclosporina	1,057 (0,664 – 1,683)	0,8
Ciclosporina + Azatioprina	1,295 (0,964 – 1,741)	0,09
Ciclosporina + Micofenolato	0,843 (0,675 – 1,054)	0,1
Micofenolato	0,998 (0,739 – 1,347)	1,0
Micofenolato + Tacrolimo	0,956 (0,426 – 2,149)	0,9
Demais esquemas	1,162 (0,692 – 1,953)	0,6
**Proporção de uso dos imunossupressores no seguimento**		
Azatioprina	1,769 (1,125 – 2,783)	0,01
Ciclosporina	1,244 (0,904 – 1,711)	0,2
Everolimo	0,051 (0,000 – 13,99)	0,3
Micofenolato	0,431 (0,311 – 0,598)	<0,001
Sirolimo	0,699 (0,199 – 2,462)	0,6
Tacrolimo	0,273 (0,092 – 0,812)	0,02

Por outro lado, atuaram como fatores protetores da sobrevivência a realização da cirurgia nas regiões Nordeste (HR = 0,688; IC 95% 0,499 – 0,950) e Sudeste (HR = 0,758; IC 95% 0,607 – 0,945); e ter maior proporção de uso dos imunossupressores micofenolato (HR = 0,431; IC 95% 0,311 – 0,598) e tacrolimo (HR = 0,273; IC 95% 0,092 – 0,812) (Tabela 4).

As causas primárias da IC e os esquemas imunossupressores de primeira escolha não apresentaram resultados significativos.

A análise multivariada demonstrou que cada ano adicional na idade do receptor, a ocorrência de infecções após o transplante e a realização do procedimento cirúrgico na região Sul estiveram relacionados ao maior risco de perda do enxerto na população estudada. Ao passo que uma maior proporção de uso dos imunossupressores micofenolato e azatioprina atuou como um fator protetor (Tabela 5). O modelo foi verificado pelo método de resíduos de Schoenfeld e demonstrou proporcionalidade de risco para todas as variáveis e correlação linear com o tempo.

**Tabela 5:** Hazard ratio para perda do enxerto: análise multivariada

Variável	HR (IC 95%)	p
Idade (ano adicional)	1,014 (1,004 – 1,025)	0,006
Ocorrência de infecções após o transplante	1,912 (1,136 – 3,243)	0,015
Região Sul	1,592 (1,240 – 2,044)	<0,001
Proporção de uso de micofenolato	0,353 (0,224 – 0,557)	<0,001
Proporção de uso de azatioprina	0,518 (0,272 – 0,988)	0,046

## Discussão

O estudo se propôs a avaliar dados pouco explorados e disseminados acerca do TC no Brasil. As análises realizadas permitem fornecer a primeira informação quanto ao tempo da sobrevivência mediana desse tipo de transplante no país, estimado em 8,3 anos entre 2000-2015.

As probabilidades de sobrevivência descritas para o primeiro (70,9%) e quinto (59,5%) anos de acompanhamento, encontram-se ligeiramente inferiores àquelas descritas pela Associação Brasileira de Transplante de Órgãos (ABTO), único órgão a publicitar tais dados no país atualmente, que informa, comparativamente, taxas de 74% e 64% para os mesmos tempos de acompanhamento.^8^ Os dados fornecidos pela ABTO, contudo, são de uma série histórica iniciada em 2010, era mais recente que a deste estudo, para a qual espera-se um aumento importante nas estimativas de sobrevivência em todo o mundo, dado o aprimoramento das equipes transplantadoras e a entrada de novos medicamentos no mercado.^9^

Dados da *International Society for Heart and Lung Transplantation* (ISHLT), demonstram que a sobrevivência mediana do TC no mundo foi de 8,6 anos no período entre 1982-1991, enquanto no período de 2002-2008 esse número alcançou 12,2 anos. As sobrevivências em um e cinco anos também se apresentam maiores que a brasileira: 81 e 69%, respectivamente. Os dados da ISHLT, entretanto, são provenientes majoritariamente de países da Europa e América do Norte, com características sociodemográficas, clínicas e sistemas de saúde bastante díspares do Brasil.^4^

Embora não tenha sido possível definir claramente as principais causas de IC, a ocorrência de cardiopatias isquêmicas como segunda causa mais relatada está em consonância com diversos estudo realizados que apontam essa como uma das principais causas da IC em todo o mundo.^9^^–^^11^ Esperava-se, no entanto, um número expressivo de registros de pacientes com doença de Chagas, dado que se trata de uma doença endêmica no país e sabidamente relacionada à ocorrência de IC, assim como de outras condições como a doença hipertensiva.^12^ Acredita-se que tal incongruência esteja associada ao fato de que o atendimento inicial desses indivíduos ocorre na atenção primária – cujos registros são escassos e não alcançados pela base de dados deste estudo – de modo que, ao chegar aos níveis de média e alta complexidade da assistência os mesmos já se encontram com um quadro de IC avançado, sendo esse seu primeiro registro.

O mesmo pode ser observado nos registros de comorbidades que não puderam ser verificados em sua totalidade. Os registros de hipertensão e dislipidemia, entretanto, fornecem um dado importante, tendo em vista que tais condições estão associadas mais comumente ao uso de ciclosporina, quando comparada com tacrolimo, mais associado ao diabetes.^13^^–^^17^ Ademais, conforme apresentado na tabela 1, o uso de ciclosporina foi significativamente maior que o de tacrolimo na população estudada. Cabe informar, contudo, que o uso de tacrolimo para o TC no Brasil ainda é *off-label*, o que impediu que o mesmo fosse disponibilizado amplamente a nível nacional até 2015, quando foi, então, incorporado pela Comissão Nacional de Incorporação de Tecnologias (CONITEC) ao *rool* de medicamentos disponíveis no SUS, juntamente com everolimo e sirolimo.^6^

Em contrapartida, as análises realizadas demonstraram que não houve diferença na efetividade dos esquemas terapêuticos utilizados. Diversos estudos corroboram tal dado, sobretudo quando se trata da comparação ciclosporina *versus* tacrolimo; ainda que alguns estudos apontem para uma menor incidência de rejeição com o tacrolimo, não há evidência de superioridade no que se refere a sobrevida dos pacientes. Na prática clínica, no entanto, observa-se um aumento expressivo no uso do tacrolimo nos últimos anos, o que poderá ocorrer também no Brasil após sua incorporação no SUS.^1^^,^^13^^–^^17^

A elevada proporção de uso de micofenolato, observado no estudo, também segue uma tendência mundial e, apesar de não ter sido verificada diferença entre as combinações terapêuticas, alguns estudos sugerem eficácia ligeiramente superior do micofenolato frente à azatioprina, assim como foi observado nas curvas de Kaplan-Meier deste estudo, embora os resultados não tenham apresentado significância estatística.^18^^–^^22^ No contexto brasileiro, é importante destacar que estudos nacionais indicam resultados desfavoráveis com o uso micofenolato em pacientes com doença de Chagas, devido a elevada incidência de reativação da doença após o transplante.^23^^–^^25^

Por outro lado, a proporção de uso de micofenolato e azatioprina mostrou-se como fator protetor da sobrevivência no modelo multivariado, sugerindo que, ainda que não haja diferença entre as estratégias terapêuticas inicialmente adotadas, o uso desses medicamentos por um período de tempo maior parece contribuir para a maior sobrevida de determinados pacientes.

Embora a proporção de uso da azatioprina tenha aparecido como fator de risco na análise univariada (Tabela 4), no modelo final a mesma surge como fator protetor, no limite da significância e muito próxima da faixa de não efeito (a saber: HR= 1,00 e p>0,05). Tal fato pode ser justificado, tendo em vista que na análise univariada são comparados os períodos de tempo de utilização dos medicamentos individualmente, logo de pacientes que utilizaram ou não apenas o medicamento em questão; já na análise multivariada, não apenas o uso da azatioprina individualmente é considerado, mas de todos os medicamentos em diferentes combinações e em conjunto com outras variáveis. Portanto, é razoável considerar que nessa condição a azatioprina não represente necessariamente um risco à sobrevivência dos pacientes, visto que os outros fatores podem oferecer maior probabilidade de óbito do que o uso do medicamento. Deve-se considerar, ainda, que grupos com características e necessidades distintas irão se beneficiar de esquemas distintos, como parece ser o caso dos pacientes chagásicos que se beneficiam do uso da azatioprina.

Ao avaliar, ainda, o uso dos esquemas terapêuticos por região, nota-se que o Sul tem maior percentual de uso da azatioprina quando comparado a todas as demais regiões, e a realização do transplante nesta região parece também influenciar a sobrevivência, de modo que a mesma figura como um fator de risco no modelo multivariado. Verificou-se ainda que o maior percentual de uso da azatioprina se dá principalmente nos anos iniciais do seguimento, entre 2000-2004; e a partir de então a proporção de uso deste medicamento no Sul aproxima-se da observada nas demais regiões. Tal dado sugere que essa diferença observada na sobrevivência entre as regiões pode estar relacionada aos protocolos de tratamento adotados nos estados do Sul do país, visto que o Brasil não dispõe de um protocolo clínico único para o TC, sobretudo no início dos anos 2000, quando o estudo e, consequentemente, as evidências de comparação entre azatioprina e micofenolato eram recentes.

Deve-se destacar, contudo, que o Brasil é um país de dimensões continentais e com grandes diferenças entre as cinco regiões, de modo que tais disparidades podem estar relacionadas ainda a outros fatores como: gravidade dos pacientes submetidos ao transplante, agilidade no transporte do órgão, estrutura física e de recursos humanos dos centros transplantadores, capacitação das equipes de transplante, além dos protocolos e diretrizes clínicas adotadas para o manejo do doador e receptor, dentre outras condições. Outros dados, portanto, são necessários para melhor elucidar todas essas condições e como elas influenciam na sobrevivência dos pacientes.

O modelo multivariado expõe, ainda, que as infecções ocorridas após o transplante e o ano de vida adicional, foram fatores de risco à sobrevivência dos indivíduos. Sabe-se que as infecções, de fato, estão entre as principais causas de morte após o TC, sobretudo no primeiro ano. Do mesmo modo, a idade do receptor está relacionada à sobrevivência, sendo observado um aumento diretamente proporcional das taxas de mortalidade, a curto e longo prazo.^1^^,^^9^

A variável demográfica sexo, reconhecidamente associada a um maior risco para a sobrevivência no TC, não se apresentou significativa no modelo final para a população estudada. No entanto, acredita-se que tal fato esteja relacionado ao tamanho significativamente distinto entre os grupos, sendo que o número de indivíduos do sexo masculino foi cerca de 2,5 vezes maior que do sexo feminino, uma vez que outros estudos sugerem sobrevida significativamente maior nas mulheres.^9^

A dificuldade em observar resultados expressivos para as variáveis clínicas, como tempo mediano de DCV antes do transplante, causa da IC e comorbidades pós transplante, relaciona-se à principal limitação deste estudo que é o uso de dados provenientes de bases administrativas. De uma maneira geral, tais bases de dados não apresentam registros de informações clínicas de forma clara e facilmente identificáveis, visto que não foram construídas com tal objetivo. Assim a avaliação de variáveis importantes relacionadas aos doadores ou à situação clínica dos pacientes antes e após o transplante e que podem influenciar diretamente na sobrevivência dos mesmos e até nas diferenças regionais observadas, não puderam ser analisadas. Além disso, as informações disponíveis podem apresentar inconsistências e omissões, decorrentes também da natureza retrospectiva do estudo.

## Conclusões

Este estudo, de âmbito nacional, apresenta dados robustos e de suma importância para a saúde pública acerca da sobrevivência de pacientes transplantados cardíacos acompanhados no SUS, potencialmente úteis na construção de diretrizes e protocolos.

A mediana de sobrevivência geral do TC no Brasil no período de 2000 a 2015 foi de 8,3 anos, ao passo que para a população de indivíduos adultos com registro de uso de imunossupressores obtidos no SUS a estimativa foi de 11,1 anos. Para esta população, o estudo demonstrou que a idade, a ocorrência de infecções após o transplante e ter realizado a cirurgia na região Sul atuaram como fatores de risco à sobrevida no período avaliado.

Tais resultados fornecem dados farmacoepidemiológicos ainda não publicados sobre o TC no Brasil, que podem ser disseminados com intuito de contribuir para a saúde pública, bem como para melhorias nas condutas adotadas e no cuidado desses pacientes.
