# Supplementary material for: Dezesseis Anos de Transplante Cardíaco em Coorte Aberta no Brasil: Análise de Sobrevivência de Pacientes em Uso de Imunossupressores
Source: Arq Bras Cardiol. 2021 Apr 8;116(4):744–53. [Article in Portuguese] doi: 10.36660/abc.20200117 (PMC8121390; doi:10.36660/abc.20200117)
Supplement: Translation

# Translation: Sixteen Years of Heart Transplant in an Open Cohort in Brazil: Analysis of Graft Survival of Patients using Immunosuppressants

**Authors:** Natália Cristina Cardoso Freitas, Mariangela Leal Cherchiglia, Charles Simão, Juliana Alvares-Teodoro, Francisco de Assis Acurcio, Augusto Afonso Guerra

**Affiliations:** 1 Belo HorizonteMG Brazil Universidade Federal de Minas Gerais - Faculdade de Farmácia - Departamento de Farmácia Social, Belo Horizonte, MG - Brazil.; 2 Belo HorizonteMG Brazil Universidade Federal de Minas Gerais - Faculdade de Medicina - Departamento de Medicina Preventiva e Social, Belo Horizonte, MG - Brazil.; 3 Belo HorizonteMG Brazil Universidade Federal de Minas Gerais - Faculdade de Medicina - Departamento de Cirurgia, Belo Horizonte, MG – Brazil.

**Keywords:** Heart Transplantation/trends, Cyclosporine/therapeutic use, Survival, Immunosuppresive Agents, Epidemiology

## Abstract

**Background:**

Heart transplant is the main therapeutic alternative for advanced heart failure patients. Several risk factors affect these patients' survival; however, few studies about the topic are available in Brazil.

**Objectives:**

To review the survival rates of heart transplant patients in the Brazilian Public Health System (Sistema Único de Saúde - SUS) between 2000 and 2015.

**Methods:**

This is a non-concurrent, open cohort study, involving cardiac transplant patients in Brazil. The cumulative survival probability was estimated by the Kaplan-Meier curve, and the curve comparison was done using the Log-Rank test. The Cox model was used to calculate the Hazard-Ratio (HR). Analyses were conducted at the 95% confidence level.

**Results:**

The heart transplant survival rate median in Brazil, during the period, was 8.3 years. Each additional year in the recipient's age, the occurrence of infections, and the performance of the surgical procedure in the South Region were associated with a higher risk of graft loss. A higher use ratio of immunosuppressants mycophenolate and azathioprine acted as a protection factor.

**Conclusions:**

The analyses conducted provide the first information about the median survival time in heart transplant patients in Brazil. The difference noticed among the geographical regions may be related to the different treatment protocols adopted in the country, especially in the early 2000s. The rate of mycophenolate and azathioprine use as a protection factor suggests that, despite the absence of differences among therapeutic strategies, use of these drugs may favor survival of certain patients. The study provides robust epidemiological data, which are relevant for public health.

## Introduction

Heart transplant (HT) is the main therapeutic alternative for patients diagnosed with advanced heart failure (HF) that is refractory to optimized clinical and surgical treatment, and its main purpose is to improve these individuals' survival and quality of life.^1^ After transplantation, the extended use of immunosuppressive therapy schemes for transplant maintenance. Although current recommendations allow for the combination and use of several drugs, triple schemes, including corticosteroids, calcineurin inhibitors and antiproliferative agents, remain widely recommended by guidelines and adopted in healthcare services.^2^

After the introduction of cyclosporine in the 1980s, the number of heart transplants and survival rates have progressively increased globally. Several risk factors, however, still affect HT survival, among which, recipient and donor demographics, clinical variables, such as HF cause, maintenance therapy strategies adopted, and the incidence of post-transplant complications.^3^^,^^4^

Brazil has one of the largest public health systems for transplant in the world, and nearly all procedures are performed by the Unified Health System (SUS). Currently, the country stands out in Latin America and it is considered to be a reference in HT in Chagas disease cases.^5^ HT and monitoring of transplanted patients, from the pre-operation procedures to the supply of post-transplant immunosuppressants, are among the thirty most expensive therapies provided to the Brazilian population by the SUS, which is responsible for approximately 96% of the HT procedures performed in the country.^6^

Unlike other countries, however, few studies on HT survival are available in Brazil. Data are scarce and diffuse and, as a result, there is no robust information regarding graft survival and its respective risk factors for the Brazilian population. In this context, the purpose of the present study is to analyze the survival of patients who underwent HT in the Brazilian SUS, between the years 2000 and 2015, with the records of immunosuppressive scheme use.

## Methods

This is a non-concurrent, open cohort study, involving patients who underwent HT in the Brazilian SUS. This cohort study was developed by means of deterministic and probabilistic record linkage – a method used to integrate and unify data from a single patient, originating from different health information systems – of the different SUS administrative data bases: SUS Hospital Information System (SIH/SUS), Outpatient Information System (SIA/SUS) and Mortality Information System (SIM).^7^ The study included patients who had undergone HT in SUS, between 01/01/2000 and 12/31/2014. The transplant record date was defined as the date the patient was added to the cohort, and a minimum 12-month monitoring period was defined, so that monitoring ended on 12/31/2015. Patients added during this first phase underwent, initially, the general survival assessment for HT in Brazil.

After that, a cohort was extracted for adult patients, to whom the following previous cohort exclusion criteria were applied: age under 18 years; individuals who underwent multiple transplantation; individuals whose first record in the cohort was that of a retransplant; and individuals for whom the database did not exhibit any records of immunosuppressant use.

### Statistical analysis

Descriptive analysis of the variables used in the study and survival analysis were conducted.

Descriptive statistical analysis was conducted for all variables. Categorical variables were analyzed by means of absolute and relative frequency distribution: gender, age group, geographical region where the transplant was conducted, primary HF diagnosis, median cardiovascular disease period prior to the transplant ≥ 17 months, comorbidities/complications developed after the transplant, and immunosuppressive therapy. Each drug use time ratio, up to the event or censoring, for each patient in the cohort, was analyzed by median and interquartile range. These measures were also presented for general age of the adult population.

Survival analyses used the following parameters: the event, defined as graft loss and represented, in this study, by the occurrence of death or retransplant; informative censoring, considered to be the date of the last record regarding immunosuppression; and right censoring, that is, study interruption represented by the monitoring end date (12/31/2015).

The Kaplan–Meier estimator was used to determine the cumulative survival probability of graft survival in patients included in both cohorts. Differences among the curves were compared by the Log-Rank test. Variables were assessed individually, to determine the effect each one of them on survival, and those that exhibited a p-value<0.20 were added to the final multivariate model. Cox's proportional hazards semi-parametric model was used to calculate the Hazard-Ratio (HR) for these univariate and multivariate analyses. Schoenfeld residuals test was used to determine the adjustment and hazard ratio in the final model. All analyses were conducted considering a 95% confidence interval.

Statistical analyses were performed using the Foundation for Statistical Computing' software “R”, version 3.6.0.

This study was approved by the Minas Gerais Federal University Committee on Research Ethics (CAAE - 16334413.9.0000.5149).

## Results

A total of 2,197 HT patients in Brazil, between 2000 and 2014, were identified, mostly males (70.7%), among which 88.9% (n=1,954) were adults, and 11.1% (n=243) were under 17 years of age. The cohort survival analysis showed rates of 70.9% (69.0 – 72.9) at one year, 59.5% (57.1 – 61.9) at five years, and reaching 45.1% (41.4 – 49.1) at ten years, and 29.1% (23.6 – 35.9%) at the end of the range (13.6 years). The HT survival rate median in the country, during the period, reached 8.3 years (Figure 1).

**Figure 1:** Graft survival of heart transplant patients in Brazil between 2000 and 2015. Note: the green and red broken lines in this figure's first chart represent, respectively, the upper and lower limits for the confidence interval (95%)

By comparing the two age groups – adults and teenagers under 17 years of age – a statistically significant difference between them is observed (p=0.003), revealing adults have a slightly lower survival rate. The same difference is observed in the comparison by gender, in which male patients exhibit a lower survival rate after HT (p=0.01).

As the main object of this study, a cohort of adult patients (over 18 years of age) was selected, initially including 1,954 patients. Among these patients, five were excluded, as they had been added to the cohort due to heart retransplant, six were excluded, as they had had multiple transplants, and 740 patients were excluded, as there were no records of medication use in the database. Among the latter, death records were identified for 456, and the remaining 284 are believed to have obtained the immunosuppressants from the supplementary healthcare system and/or at their own expenses. Therefore, 1,203 patients were included in the study.

Median survival rate for this population – adult patients using immunosuppressive schemes – was 11.1 years. Survival rates at one, five and ten years were 89.8% (88.1 – 91.6), 75.9% (73.1 – 78.8) and 57.0% (52.1 – 62.3), respectively.

Among the 1,203 patients included in the study, the majority was male (73.2%), with an average age of 48 years (38 – 56). For 69.1% of these patients (n=831), it was not possible to identify exactly the primary condition that led to the onset of HF, as the first record in the database was the condition itself. Ischemic cardiopathies appear as the second most frequently reported cause, corresponding to 14.1%, while other causes and congenital malformations were the least frequent causes, corresponding to 0.3 and 1.7 of the records, respectively (Table 1).

**Table 1:** Demographics of the study population

Characteristics	Total (n = 1203)	
	n	%
**Geographical region where transplant was performed**		
Central-West	43	3.6
Northeast	222	18.5
North	8	0.7
Southeast	672	55.9
South	258	21.4
**Gender**		
Female	323	26.8
Male	880	73.2
**Age group (years of age)**		
18 - 25 years of age	54	4.5
26 - 35 years of age	179	14.9
36 - 45 years of age	271	22.5
46 - 55 years of age	392	32.6
56 - 65 years of age	278	23.1
> 65 years of age	29	2.4
**Causes of heart failure**		
Cardiomyopathies	76	6.3
Undefined cardiomyopathies	831	69.1
Ischemic cardiomyopathies	170	14.1
Congenital malformations	20	1.7
Other cardiac conditions	4	8.5
Other causes	102	0.3
**Median period with previous cardiovascular disease**		
Median time lower than or equal to 17 months	434	36.1
Median time greater than 17 months	427	35.5
**Comorbidities/post-transplant complications**		
Dyslipidemia	48	4.0
Arterial hypertension	134	11.1
Infections	45	3.7
Neoplasia	11	0.9
**Events**		
Censoring	891	74.1
Death	307	25.5
Retransplant	5	0.4

Few records were checked for the comorbidities that took place after transplant, among which: arterial hypertension (11.1%), infections (3.7%), dyslipidemia (4.0%), and neoplasia (0.9%) (Table 1). No records were found for diabetes, chronic renal failure, or osteoporosis.

Most of the transplant surgeries were performed in the Southeast region (55.9%), South (21.5%) and Northeast (18.5%) (Table 1), and statistically significant differences were observed in the survival rates of patients subjected to the procedure in these regions. The Northeast and Southeast regions exhibited higher survival rates (p= 0.02 and p= 0.01, respectively), while the South region exhibited rates lower than the national average (p<0.0001). The Central-West and North regions have not exhibited significant differences (Figure 2).

**Figure 2:** Graft survival of adult heart transplant patients in Brazil between 2000 and 2015 by region.

Among the different immunosuppressive schemes, the use of cyclosporine associated with mycophenolate was the most frequently first-choice therapy scheme used (58.4%), followed using mycophenolate in monotherapy (18.4%), and by the association between cyclosporine and azathioprine (11.9%). The use of tacrolimus as a calcineurin inhibitor as first line treatment was incipient during this period, as only 3.3% of the individuals started their treatment with it, whereas mycophenolate was the most frequently used antiproliferative agents, being present in approximately 81% of the therapy schemes (Table 2).

**Table 2:** First choice immunosuppressant schemes used by the study population

Main immunosuppressant schemes Start of cohort	N	%	%accumulated*
Cyclosporine + Mycophenolate	702	58.4%	58.4%
Mycophenolate (monotherapy)	221	18.4%	76.7%
Cyclosporine + Azathioprine	143	11.9%	88.6%
Cyclosporine (monotherapy)	52	4.3%	92.9%
Mycophenolate + Tacrolimus	34	2.8%	95.8%
**Subtotal**	**1152**	**95.8%**	**95.8%**
**Other immunosuppressant schemes Start of cohort**			
Azathioprine (monotherapy)	22	1.8%	97.6%
Mycophenolate + Sirolimus	15	1.2%	98.8%
Tacrolimus (monotherapy)	3	0.2%	99.1%
Azathioprine + Cyclosporine + Mycophenolate	2	0.2%	99.3%
Azathioprine + Tacrolimus	2	0.2%	99.4%
Cyclosporine + Sirolimus	2	0.2%	99.6%
Sirolimus (monotherapy)	2	0.2%	99.8%
Azathioprine + Sirolimus	1	0.1%	99.8%
Mycophenolate + Cyclosporine + Sirolimus	1	0.1%	99.9%
Mycophenolate + Sirolimus + Tacrolimus	1	0.1%	100.0%
**Subtotal**	**51**	**4.2%**	**100%**
**Total**	**1203**	**100%**	**100%**

* sum of each scheme percentage line by line.

Stratification of the use of first choice immunosuppressive schemes by region allowed for observing that the use of cyclosporine and azathioprine was proportionally higher in the country's South region (27.9%), corresponding to approximately 2.3 times the national average. Nevertheless, the association of cyclosporine and mycophenolate was the most frequently used therapy scheme in all regions (Table 3).

**Table 3:** First choice immunosuppressant schemes used by the study population stratified by geographical region

Transplant Center Region	Azathio + Cyclos	Cyclos (monotherapy)	Cyclos + Mycophe	Mycophe (monotherapy)	Mycophe + Tacrol	Other schemes	Overall total
	n (%)						
Central-West	5(11.6)	1(2.3)	17(39.5)	8(18.6)	1(2.3)	11(25.6)	43(100.0)
Northeast	12(5.4)	7(3.1)	133(59.9)	58(26.1)	1(0.4)	11(4.9)	222(100.0)
North	0(0.0)	0(0.0)	8(100.0)	0(0.0)	0(0.0)	0(0.0)	8(100.0)
Southeast	54(8.0)	18(2.7)	410(61.0)	138(20.5)	28(4.2)	24(3.6)	672(100.0)
South	72(27.9)	26(10.1)	134(51.9)	17(6.6)	4(1.5)	5(1.9)	258(100.0)
Overall total	143(11.9)	52(4.3)	702(58.4)	221(18.4)	34(2.8)	51(4.2)	1203(100.0)

Azathio: azathioprine; Cyclos: cyclosporine; Mycophe: mycophenolate; Tacrol: tacrolimus.

No statistically significant differences (p=0.6) were observed upon evaluating patient survival based on the immunosuppressive scheme initially used (Figure 3).

**Figure 3:** Graft survival of adult heart transplant patients in Brazil between 2000 and 2015 by immunosuppressive scheme.

The median of immunosuppressant use over the period was 83.3% for mycophenolate (65.7 – 95.2), 71.1% for cyclosporine (38.5 – 91.7), 38.2% for azathioprine (11.5 – 66.8), 26.0% for tacrolimus (8.3 – 47.2), 15.0% for sirolimus (4.8 – 34.7) and 7.1% for everolimus (2.4 – 28.8).

Univariate analysis of potential risk factors for graft survival revealed a higher risk associated with male patients (HR = 1.342; CI 95% 1.02 – 1.767), with an additional year in the recipient's age (HR = 1.01; CI 95% 1.003 – 1.023), with the surgery being performed in the South region of Brazil (HR = 1.784; CI 95% 1.407 – 2.262), with the median cardiovascular (CVD) time prior to the transplant being higher than 17 months (HR = 1.389; CI 95% 1.067 – 1.807), with the development of post-transplant infections (HR = 1.702; CI 95% 1.012 – 2.861), and with a higher ratio of azathioprine use during the monitoring period (HR = 1.769; CI 95% 1.125 – 2.783) (Table 4).

Conversely, the following acted as survival protection factors, surgeries being performed in the Northeast (HR = 0.688; CI 95% 0.499 – 0.950) and Southeast (HR = 0.758; CI 95% 0.607 – 0.945) regions; and having a higher ratio of mycophenolate (HR = 0.431; CI 95% 0.311 – 0.598) and tacrolimus (HR = 0.273; CI 95% 0.092 – 0.812) use (Table 4).

**Table 4:** Graft loss hazard ratio - univariate analysis

Variable	Total (n = 1203)	
	HR (CI 95%)	p
**Geographical region where transplant was performed**		
Central-West	1.128 [0.580 - 2.194]	0.7
Northeast	0.688 [0.499 - 0.950]	0.02
North	1.489 [0.555 - 3.997]	0.4
Southeast	0.758 [0.607 - 0.945]	0.01
South	1.784 [1.407 - 2.262]	<0.001
Gender, Male	1.342 [1.019 - 1.767]	0.04
Age	1.013 [1.003 - 1.023]	0.01
**Causes of heart failure**		
Cardiomyopathies	0.962 [0.617 - 1.498]	0.9
Undefined cardiomyopathies	1.144 [0.899 - 1.457]	0.3
Ischemic cardiomyopathies	0.950 [0.681 - 1.323]	0.8
Congenital malformations	0.349 [0.087 - 1.404]	0.1
Other cardiac conditions	3.67 [0.912 - 14.77]	0.05
Other causes	0.863 [0.593 - 1.256]	0.4
Median CVD time prior to transplant	1.389 [1.067 - 1.807]	0.01
**Onset of post-transplant comorbidities**		
Dyslipidemia	0.919 [0.473 - 1.786]	0.8
Arterial hypertension	1.270 [0.896 - 1.800]	0.2
Infections	1.702 [1.012 - 2.861]	0.04
Neoplasia	1.363 [0.339 - 5.490]	0.7
**First choice immunosuppressant schemes**		
Cyclosporine	1.057 [0.664 - 1.683]	0.8
Cyclosporine + Azathioprine	1.295 [0.964 - 1.741]	0.09
Cyclosporine + Mycophenolate	0.843 [0.675 - 1.054]	0.1
Mycophenolate	0.998 [0.739 - 1.347]	1.0
Mycophenolate + Tacrolimus	0.956 [0.426 - 2.149]	0.9
Other schemes	1.162 [0.692 - 1.953]	0.6
**Ratio of immunosuppressant use in the segment**		
Azathioprine	1.769 [1.125 - 2.783]	0.01
Cyclosporine	1.244 [0.904 - 1.711]	0.2
Everolimus	0.051 [0.000 - 13.99]	0.3
Mycophenolate	0.431 [0.311 - 0.598]	<0.001
Sirolimus	0.699 [0.199 - 2.462]	0.6
Tacrolimus	0.273 [0.092 - 0.812]	0.02

Primary HF causes and the first-choice immunosuppressant schemes exhibited significant results.

Multivariate analysis showed that each additional year in the recipient's age, the occurrence of infections after the transplant, and the performance of the surgical procedure in the South region were associated with a higher risk of graft loss. However, a higher use ratio of immunosuppressants mycophenolate and azathioprine acted as a protection factor (Table 5). The model was verified by the Schoenfeld residuals method, and it demonstrated a risk proportionality for all variables, as well as linear correlation to time.

**Table 5:** Graft loss hazard ratio: multivariate analysis

Variable	HR (CI 95%)	p
Age (additional year)	1.014 [1.004 - 1.025]	0.006
Post-transplant infections	1.912 [1.136 - 3.243]	0.015
South Region	1.592 [1.240 - 2.044]	<0.001
Mycophenolate use ratio	0.353 [0.224 - 0.557]	<0.001
Azathioprine use ratio	0.518 [0.272 - 0.988]	0.046

## Discussion

The study is designed to evaluate underexplored and disseminated data about HT in Brazil. Analyzes performed allow for providing initial information about the median survival time for this type of transplant in the country, estimated at 8.3 years, between 2000 and 2015.

Survival probabilities described for the first (70.9%) and the fifth (59,5%) years of monitoring, are slightly lower than those described by the Brazilian Association of Organ Transplantation (ABTO), the only agency that currently publicizes such data in the country, which provides, comparatively, the rates of 74% and 64% for the same monitoring times.^8^ Data provided by ABTO, however, come from a historical series started in 2010; therefore, more recent than the one used in this study, for which an important increase is expected for survival estimates worldwide, considering the improvement of transplantation teams and the arrival of new drugs in the market.^9^

Data from the International Society for Heart and Lung Transplantation (ISHLT) show that median HT survival worldwide was 8.6 years in the period between 1982 and 1991, whereas in the period between 2002 and 2008 this number reached 12.2 years. Survival rates at one and five years are also higher than the Brazilian rates: 81 and 69%, respectively. ISHLT data, however, originate primarily from European and North American countries, which have quite different sociodemographic and clinical characteristics, as well as the health systems, from those in Brazil.^4^

Although it was impossible to clearly define the main HF causes, the occurrence of ischemic cardiomyopathies as the second most frequent cause is in agreement with several studies performed that indicate this as one of the main HF causes worldwide.^9^^–^^11^ A significant number of Chagas disease patient records was expected, given this is an endemic disease in the country and it is known to be related to the occurrence of HF. Other conditions, such as hypertensive disease, were also expected.^12^ Such inconsistency is believed to be associated with the fact that early treatment of these patients takes place at primary health care centers - whose records are scarce and are not reached by this study's database - so that, when medium and high complexity assistance levels are reached, patients face advanced HF, and this is their first record.

The same applies to comorbidity records that could not have been checked in full. Hypertension and dyslipidemia records, however, provide important data, as such conditions are commonly associated with the use of cyclosporine, when compared to tacrolimus, more frequently associated with diabetes.^13^^–^^17^ In addition, as provided in table 1, the use of cyclosporine was significantly greater than the use of tacrolimus in the studied population. The use of tacrolimus for HT in Brazil, it should be noted, however, is still done off-label, and this prevented this drug from being widely available at the national level until 2015, when it was added, by the National Committee for Health Technology Incorporation (CONITEC) to the list of drugs provided by the SUS, along with everolimus and sirolimus.^6^

Conversely, the analyses conducted demonstrated that no differences in effectiveness have been detected among the therapy schemes used. Several studies corroborate these data, especially in relation to the comparison between cyclosporine and tacrolimus; although some studies indicate a lower occurrence of rejection when tacrolimus is used, there is no evidence of its superiority for patient survival purposes. In clinical practice, however, there has been a significant increase in the use of tacrolimus for the past years, which may also take place in Brazil after its addition to the list of drugs provided by the SUS.^1^^,^^13^^–^^17^

The high rates of mycophenolate use observed in the study also follow a global trend and, although no differences were detected among the therapeutic combinations, some studies suggest mycophenolate has a slightly superior effectiveness in relation to azathioprine, as observed in the Kaplan-Meier curves presented in this study, despite the absence of statistically significant results.^18^^–^^22^ In the Brazilian context, it is noteworthy that national studies indicate unfavorable results with the use of mycophenolate in Chagas disease patients, due to the high rates of disease reactivation after transplantation.^23^^–^^25^

Nevertheless, the rate of mycophenolate and azathioprine use has proved to be a survival protection factor in the multivariate model, suggesting that, despite the absence of differences among therapeutic strategies used initially, the use of these drugs for a longer period, appears to contribute to the survival of certain patients.

Although the rate of azathioprine use appeared as a risk factor in the univariate analysis (Table 4), it appears as a protection factor in the final model, within the significance limit and close to the ineffective range (namely: HR= 1.00 and p>0.05). This fact may be justifiable, considering that, in univariate analysis, medication use periods are compared individually, that is, whether patients have used the medication in question or not. In multivariate analysis, however, the use of azathioprine is considered individually, as well as the use of all drugs in different combinations and along with other variables. Therefore, it is reasonable to consider that, under these conditions, azathioprine does not necessarily represent a risk to patient survival, considering that other factors may pose higher death risks than the medication use. The fact that groups with different characteristics and needs will benefit from different schemes must be also taken into account, as this appears to be the case of Chagas disease patients, who benefit from azathioprine use.

Furthermore, upon assessing the use of therapy schemes by geographical region, the South region exhibits a higher azathioprine use percentage when compared to all other regions. In addition, transplantation procedures being performed in this region also appear to affect survival, resulting in its characterization as a risk factor in the multivariate model, Higher azathioprine use percentage was also observed mainly in the first years of the monitoring period, between 2000 and 2004. From then on, this drug use rate in the South region is close to the rate observed in other geographical regions. These data suggest that the difference observed in survival rates among the geographical regions may be related to treatment protocols adopted in the South region, considering that Brazil does not have a unified clinical protocol for HT, mainly during the early 2000s, when the study and, consequently, evidence of comparison between azathioprine and mycophenolate were recent.

Brazil is notably a country of continental proportions with significant differences among its five geographical regions; therefore, these discrepancies may also be related to other factors, such as, illness severity of patients subject to transplantation, agility in organ transportation, physical and human resource structure in the transplantation centers, transplantation team qualification, in addition to clinical guidelines and protocols adopted for handling donor and recipient, among other conditions. Other data, therefore, are required to clarify all of these conditions, as well as how they affect patient survival.

The multivariate model also showed that infections occurred after transplantation and the additional year of age were risk factors to patient survival. Infections are known to be one of the main causes of death after HT, especially during the first year. Similarly, recipient age is related to survival, and a directly proportional increase in mortality rates is observed in short and long terms.^1^^,^^9^

The ‘gender’ demographic variable, admittedly associated with higher risk for survival in HT, was not significant in the final model for the studied population. Nevertheless, this fact is believed to be associated with a significant difference in size among the groups, as the number of male patients was approximately 2.5 times the number of female patients, considering that other studies suggest significantly higher survival rates in women.^9^

Difficulties in observing relevant results for clinical variables, such as median CVD time prior to the transplant, HF cause and post-transplantation comorbidities, are related to the main limitation in this study, which is the use of data originating from administrative databases. In general, such databases provide no clear and easily identifiable records of clinical information, as they were not built for these purposes. Therefore, the assessment of important variables related to donors or to the patients' clinical condition before and after transplantation, and which may directly affect their survival rates or the regional differences observed, could not be reviewed. In addition, information available may exhibit inconsistencies and omissions, also due to the retrospective nature of the study.

## Conclusions

This study, with a nationwide reach, presents robust data, which have great relevance for the public health system, about the survival of HT patients monitored by the SUS, potentially useful for the development of guidelines and protocols.

The general survival rate median for HT patients in Brazil, between 2000 and 2015, was 8.3 years, whereas for adult individuals with records of using immunosuppressant provided by the SUS, the estimated survival period was 11.1 years. For this population, the study demonstrated that age, the occurrence of infection after transplantation, and having had surgery in the South region acted as risk factors to survival in the period studied.

These results provide unpublished epidemiological data on HT in Brazil, which may be publicized to contribute with the public health system, as well as with the conduct adopted and for these patients' care.
